# Supplementary material for: Amphibian pore-forming protein βγ-CAT drives extracellular nutrient scavenging under cell nutrient deficiency
Source: iScience. 2023 Apr 7;26(5):106598. doi: 10.1016/j.isci.2023.106598 (PMC10148134; doi:10.1016/j.isci.2023.106598)

iScience, Volume *26*

Supplemental information

Amphibian pore-forming protein βγ-CAT drives extracellular nutrient scavenging under cell nutrient deficiency

Ling-Zhen Liu, Long Liu, Zhi-Hong Shi, Xian-Ling Bian, Zi-Ru Si, Qi-Quan Wang, Yang Xiang, and Yun Zhang

Supplemental Figure Legends

Supplemental Figure 1. The proportion of hepatocytes and liver glycogen in isolated toad liver cells was detected. (A) The proportion of $CK18^{+}$ hepatocytes was determined by flow cytometry with an anti-cytokeratin 18 (CK18, a hepatocyte marker) antibody. (B) The hemolytic activity of exogenous βγ-CAT (5 nM) was assayed in PBS, $Glc^{+}/Gln^{+}/Pyr^{+}$, $Glc^{-}/Gln^{-}/Pyr^{-}$, or $Glc^{+}/Gln^{-}/Pyr^{-}$ medium, respectively. (C) The hemolytic activity of liver cell supernatants under $Glc^{-}/Gln^{-}/Pyr^{-}$ conditions was detected in the presence of anti-βγ-CAT antibodies (100 μg/mL), anti-BmTFF3 antibodies (200 μg/mL), or rabbit IgG (an antibody control). (D) Toad liver cells were cultured in $Glc^{+}/Gln^{+}/Pyr^{+}$ or $Glc^{-}/Gln^{-}/Pyr^{-}$ medium for 1, 3 and 5 hours, respectively. intracellular glycogen levels in the cells were detected by colorimetric assay. Data (B–D) are represented as the mean ± SD of triplicate samples. ns ($p \geq 0.05$), ***$p<0.001$ and ****$p<0.0001$ by two-way ANOVA (B,D) or by one-way ANOVA (C). All data are representative of at least two independent experiments. Related to figure 1.

Supplemental Figure 2. Compound C and SBI-0206965 inhibit activation of AMPK signaling in toad liver cells under $Glc^{-}/Gln^{-}/Pyr^{-}$ conditions. (A) Sequence alignment of AMPKs (*Top*) and ACCs (*Bottom*) in toad *B. maxima* and other species was analyzed by Clustal Omega6. The activation loop of AMPK is indicated by a green line. The phosphorylated site (Thr residue of AMPKs and Ser residue of ACCs) is indicated by a red spot. (B) AMPK signaling activation in toad liver cells after culture in $Glc^{+}/Gln^{+}/Pyr^{+}$, $Glc^{-}/Gln^{-}/Pyr^{-}$, or $Glc^{+}/Gln^{-}/Pyr^{-}$ medium for 3 hours was detected by Western blotting. (C, D) Cytotoxicity of compound C (C) and SBI-0206965 (D) in toad liver cells was detected by PI staining after treatment with compound C or SBI-0206965 in $Glc^{-}/Gln^{-}/Pyr^{-}$ medium for 3 hours. (E) Phosphorylation of pS79-ACC1 in toad liver cells was determined by Western blotting after treatment with compound C or SBI-0206965 in $Glc^{-}/Gln^{-}/Pyr^{-}$ medium for 3 hours. Data (C, D) are represented as the mean ± SD of triplicate samples. ns

($p \geq 0.05$) by one-way ANOVA. All data are representative of at least two independent experiments. Related to figure 2.

Supplemental Figure 3. βγ-CAT promotes extracellular protein intake under cell nutrient deficiency. (A) the LDH release of toad liver cells was detected by an LDH cytotoxicity assay kit after the cells cultured with or without βγ-CAT for 3 hours. (B) The mean fluorescence intensity of Ovalbumin-DQ in HepG2 cells was determined by flow cytometry after treatment with 40 nM βγ-CAT and 20 μg/mL Ovalbumin-DQ in $Glc^-/Gln^-/Pyr^-$ medium for 30 minutes. (C, D) Inhibitory effect of a macropinocytosis inhibitor (EIPA) on protein intake induced by βγ-CAT. Toad liver cells (C) and HepG2 cells (D) were incubated with or without 100 μM EIPA in $Glc^-/Gln^-/Pyr^-$ medium for 1 hour. Then, the cells were incubated with 100 μg/mL FITC-OVA in the presence of 100 nM βγ-CAT (toad liver cells) or 40 nM βγ-CAT (HepG2 cells) for 30 minutes. The mean fluorescence intensity of Ovalbumin-DQ in toad liver cells and HepG2 cells was determined by flow cytometry. (E) The mean fluorescence intensity of Bm-SA in toad liver cells was determined by flow cytometry after treatment with 100 nM βγ-CAT or 100 μg/mL anti-βγ-CAT antibodies in $Glc^-/Gln^-/Pyr^-$ medium for 30 minutes. (F) The intracellular content of representative amino acids in toad liver cells was determined by LC-MS and LC-MS/MS after incubation with 500 μg/mL BSA and 100 nM βγ-CAT in $Glc^-/Gln^-/Pyr^-$ medium for 7 hours. (G) The ATP content in toad liver cells was determined by an ATP detection kit after incubation with 500 μg/mL BSA and 100 nM βγ-CAT or 100 μg/mL anti-βγ-CAT antibodies in $Glc^-/Gln^-/Pyr^-$ medium for 7 hours. Rabbit IgG was used as an antibody control. Results are reported as the mean ± SD of triplicate samples, ns ($p \geq 0.05$), *$p<0.05$, **$p<0.01$, ***$p<0.001$, and ****$p<0.0001$ by the one-way ANOVA. All data are representative of at least two independent experiments. Related to figure 3.

Supplemental Figure 4. Viability of toad liver cells and cytotoxicity of βγ-CAT in HepG2 cells under $Glc^-/Gln^-/Pyr^-$ conditions. (A) The viability of toad liver cells

was determined by PI staining after culture in $Glc^{+}/Gln^{+}/Pyr^{+}$, $Glc^{-}/Gln^{-}/Pyr^{-}$, or $Glc^{-}/Gln^{-}/Pyr^{-}$ plus βγ-CAT (100 nM) for 0–11 hours. (B) Cytotoxicity of βγ-CAT in HepG2 cells was detected by MTS assays after treatment with various concentrations of βγ-CAT in $Glc^{-}/Gln^{-}/Pyr^{-}$ medium for 36 hours. Results are reported as the mean ± SD of triplicate samples. In (A), statistical significance was calculated by two-way ANOVA, $^{**}p<0.01$, and $^{****}p<0.0001$ ($Glc^{-}/Gln^{-}/Pyr^{-}$ vs. $Glc^{+}/Gln^{+}/Pyr^{+}$ groups) and ns $p\geq0.05$ ($Glc^{-}/Gln^{-}/Pyr^{-}$ + βγ-CAT vs. $Glc^{-}/Gln^{-}/Pyr^{-}$ groups). In (B) ns ($p\geq0.05$), $^{*}p<0.05$, and $^{***}p<0.001$ by one-way ANOVA. All data are representative of at least two independent experiments. Related to figure 4.

Supplemental Figure 1

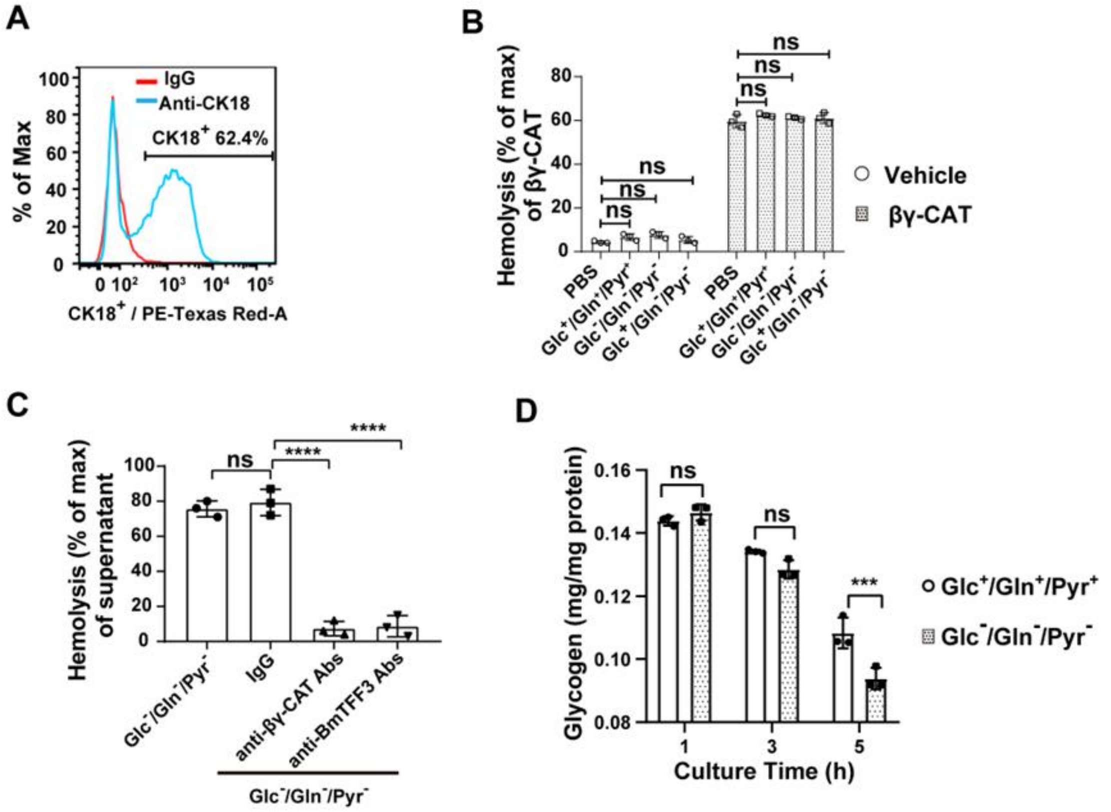

Supplemental Figure 2

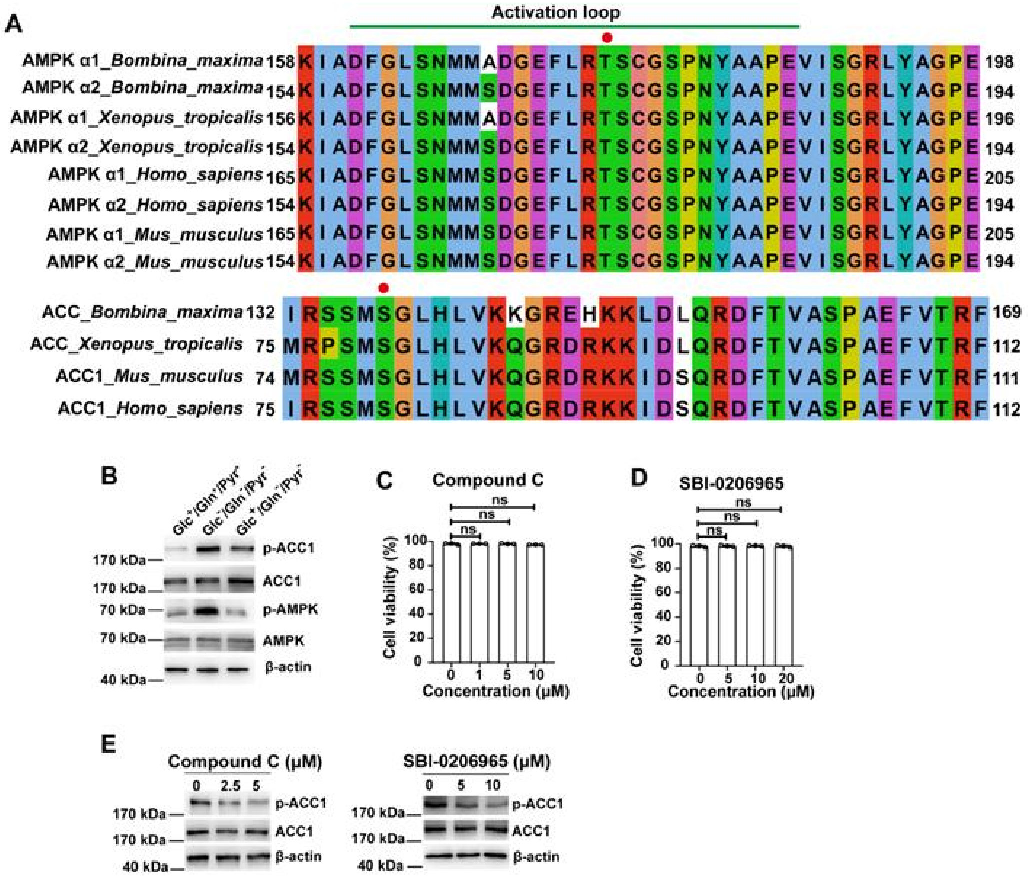

Supplemental Figure 3

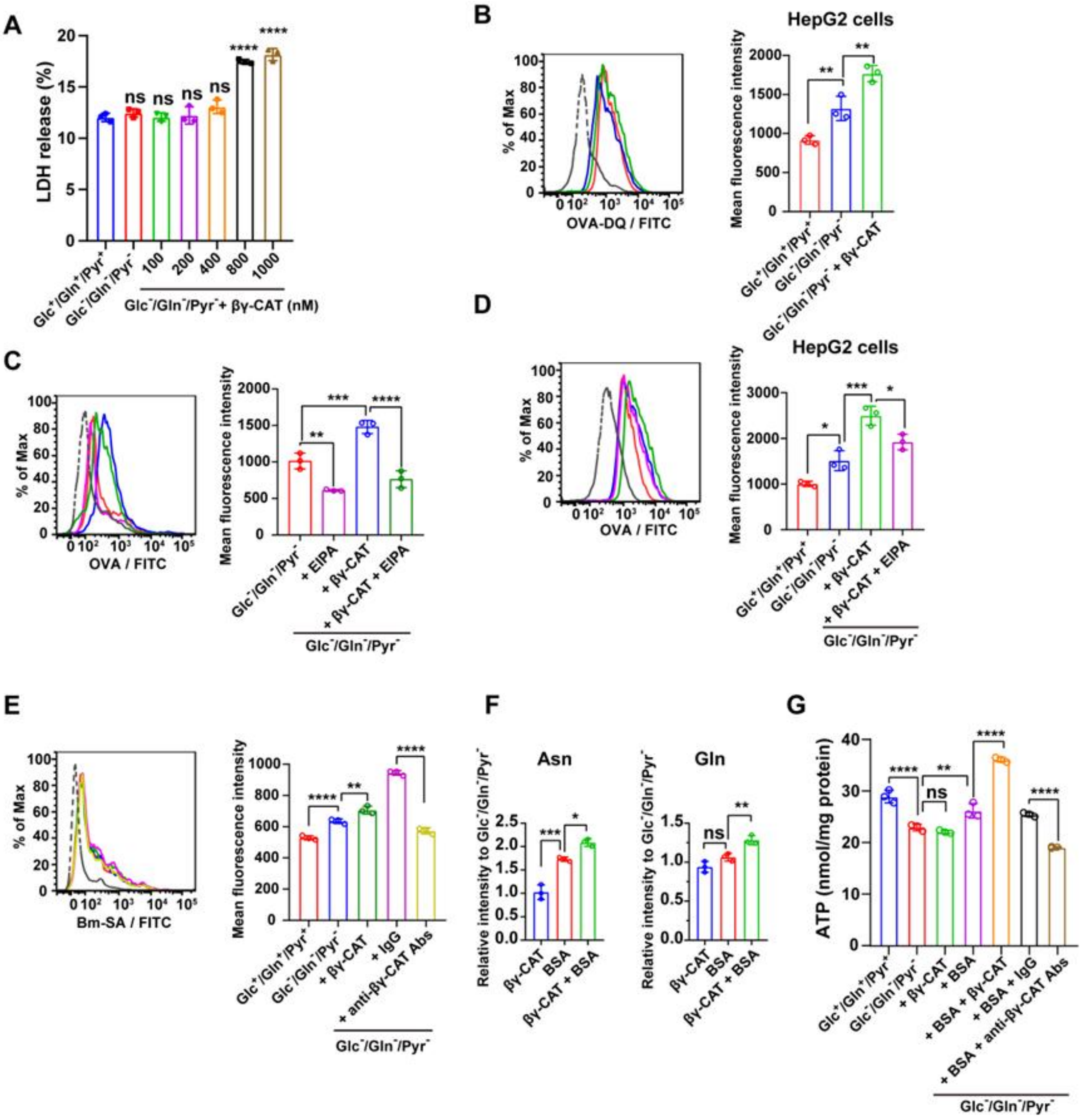

Supplemental Figure 4

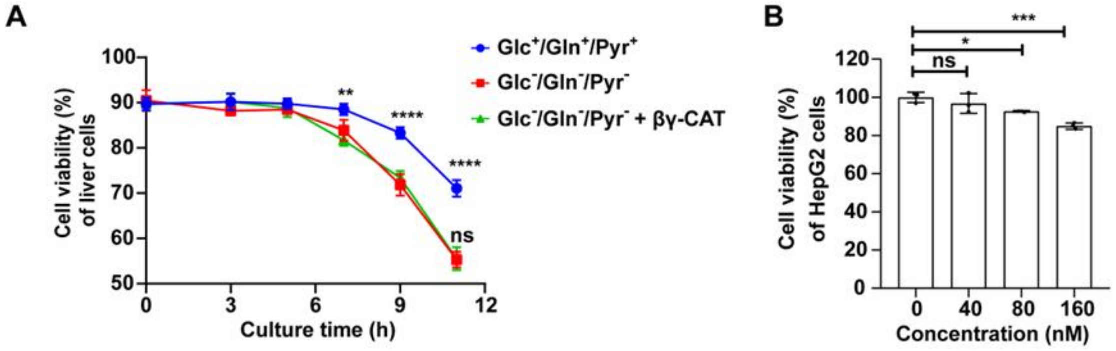

Supplement: Document S1. Figures S1–S4 [file mmc1.pdf]
